# Consensus Recommendations for the Diagnosis, Biomarker Testing, and Clinical Management of Advanced or Metastatic Non-small Cell Lung Cancer With Mesenchymal-Epithelial Transition Exon 14 Skipping Mutations in the Middle East, Africa, and Russia

**DOI:** 10.7759/cureus.41992

**Published:** 2023-07-17

**Authors:** Mervat Mahrous, Abdalla Omar Jebriel, Ahmed Allehebi, Amr Shafik, Fadi El Karak, Filippo Venturini, Hamed Alhusaini, Matthias Meergans, Mehmet Ali Nahit Sendur, Mohamed Ouda, Muath Al-Nassar, Saadettin Kilickap, Saeed Al Turki, Turki Al-Fayea, Yasser Abdel Kader

**Affiliations:** 1 Oncology, Minia University, Minia, EGY; 2 Oncology, Prince Sultan Military Medical City, Riyadh, SAU; 3 Oncology, School of Medicine, Misurata University, Misurata, LBY; 4 Oncology, King Faisal Specialist Hospital & Research Center, Jeddah, SAU; 5 Oncology, Faculty of Medicine, Ain Shams University, Cairo, EGY; 6 Oncology, Saint Joseph University of Beirut, School of Medicine, Beirut, LBN; 7 Oncology, Hôtel-Dieu de France, Beirut, LBN; 8 Oncology, Clemenceau Medical Center, Dubai, ARE; 9 Oncology, Merck Serono S.p.A., Rome, ITA; 10 Oncology, King Faisal Specialist Hospital & Research Center, Riyadh, SAU; 11 Oncology, Merck Serono Middle East FZ-Ltd., Dubai, ARE; 12 Oncology, Ankara Yıldırım Beyazıt University, Faculty of Medicine, Ankara, TUR; 13 Oncology, Kuwait Cancer Control Center, Kuwait City, KWT; 14 Medical Oncology, Istinye University, Faculty of Medicine, Ankara, TUR; 15 Oncology, Anwa Labs, Riyadh, SAU; 16 Oncology, King Fahad Medical City - Ministry of National Guard, Jeddah, SAU; 17 Oncology, King Saud Bin Abdulaziz University for Health Sciences, Jeddah, SAU; 18 Oncology, Faculty of Medicine, Cairo University, Cairo, EGY

**Keywords:** non-small-cell lung cancer, targeted therapy, overall survival, next-generation sequencing, met tyrosine kinase inhibitors, met exon 14 skipping mutation, lung cancer, liquid biopsy, immunotherapy, chemotherapy

## Abstract

Mesenchymal-epithelial transition exon 14 (METex14) skipping mutations occur in about 3%-4% of patients with non-small cell lung cancer (NSCLC). This is an aggressive subtype associated with poor prognosis. METex14 skipping is a potentially targetable mutation. Targeted therapy is a promising treatment modality for patients with advanced/metastatic METex14-mutant NSCLC. Performing systematic molecular testing to detect the driver mutation is essential for initiating targeted therapy. However, there is a lack of guidelines on molecular testing for assessing the eligibility of patients for targeted therapy. Therefore, a multidisciplinary panel consisting of experts from the Middle East, Africa, and Russia convened via a virtual advisory board meeting to provide their insights on various molecular testing techniques for the diagnosis of METex14 skipping mutation, management of patients with targeted therapies, and developing consensus recommendations for improving the processes. The expert panel emphasized performing molecular testing and liquid biopsy before treatment initiation and tissue re-biopsy for patients with failed molecular testing. Liquid biopsy was recommended as complementary to tissue biopsy for disease monitoring and prognosis. Selective MET inhibitors were recommended as the first and subsequent lines of therapy. These consensus recommendations will facilitate the management of METex14 skipping NSCLC in routine practice and warrant optimum outcomes for these patients.

## Introduction and background

Lung cancer is a prominent, highly metastasizing malignancy responsible for cancer-related mortality worldwide [1]. Globally, lung cancer is the second most frequent cancer (11.4%) and a major contributor to cancer-related deaths (18%) [2]. In the Middle East and North Africa region, lung cancer remains the most common type of cancer, and 79,887 people were diagnosed with lung cancer in 2018 [3]. The incidence of lung cancer in Saudi Arabia was 3.8% with a mortality rate of 7.4% per the 2018 estimates of the World Health Organization (WHO) [4]. In 2020, lung cancer was ranked among the top 10 cancers in Arab-world females [5]. An epidemiological study based on the Saudi Cancer Registry reported 4,530 lung cancer cases from 2006 to 2016 and the age-standardized incidence ratio was 3.2 [1]. With an overall frequency of 17.3%, lung cancer continues to be the most frequently diagnosed cancer in Turkey, and the foremost cause of cancer mortality, resulting in 23.9% of all cancer-related deaths [6].

Non-small cell lung cancer (NSCLC) is the most common type of lung cancer constituting around 85% of all lung cancers [7]. It is a debilitating subtype of lung cancer and is the prominent cause of death associated with cancer [8,9]. Several oncogenic driver gene mutations, including epidermal growth factor receptor (EGFR), Kirsten rat sarcoma viral oncogene homolog (KRAS), anaplastic lymphoma kinase (ALK), mesenchymal-epithelial transition (MET), c-ROS oncogene 1 (ROS1), and rearranged during transfection (RET) are known to play an essential role in the development of NSCLC [10]. Various clinical trials on targetable driver mutations in patients with NSCLC are shown in Table 1.

**Table 1:** Clinical trials on targetable driver mutations in patients with NSCLC. ALK: anaplastic lymphoma kinase; BID: twice daily; EGFR: epidermal growth factor receptor; KRAS: Kirsten rat sarcoma viral oncogene homolog; NSCLC: non-small cell lung cancer; OD: once daily; RET: rearranged during transfection; ROS1: c-ROS oncogene

Trial name, phase	Driver mutations in patient population (n)	Drug/dose	Response rate (%)
ARROW, phase I/II [11]	RET fusion-positive advanced or metastatic NSCLC and ECOG PS 0-2 (n = 233)	Pralsetinib, 400 mg OD	Overall response: 61% (95% CI = 50 to 71)
NCI-MATCH, ongoing trial with open sub-protocols [12]	ALK (n = 5) or ROS1 rearrangements (n = 4)	Crizotinib, 250 mg BID, 28-day cycle	Sub-protocol F (ALK): 50% (90% CI = 9.8 to 90.2); sub-protocol G (ROS1): 25% (90% CI = 1.3 to 75.1)
FLAURA, Phase III [13]	EGFR-positive advanced NSCLC (n = 556)	Osimertinib (80 mg OD) or either gefitinib (250 mg OD) or erlotinib (150 mg OD)	Median overall survival: osimertinib group: 38.6 months (95% CI = 34.5 to 41.8); comparator group: 31.8 months (95% CI = 26.6 to 36.0)
CROWN, Phase III [14]	ALK-positive advanced NSCLC (n = 296)	Lorlatinib, 100 mg OD or crizotinib 250 mg BID, 28 days cycle	Objective response: lorlatinib group: 76% (95% CI = 68 to 83); crizotinib group: 58% (95% CI = 49 to 66)
Phase II [15]	KRAS p.G12C-mutated advanced NSCLC (n = 126)	Sotorasib, 960 mg OD	Objective response: 37.1% (95% CI = 28.6 to 46.2)

Approximately 35%-50% of patients with non-squamous NSCLC have been estimated to undergo targetable mutations [16]. MET is a receptor tyrosine kinase (TK) and its natural ligand is the hepatocyte growth factor (HGF). MET exon 14 (METex14) skipping mutation occurs due to somatic mutations in the MET gene resulting in the production of an incomplete MET receptor without a tyrosine binding site. This, in turn, causes activation of the MET signaling pathway with elevated oncogenic potential [17]. The prevalence of METex14 skipping mutation is approximately 3%-4% of NSCLC cases [18]. In patients with squamous and sarcomatoid NSCLC, there is approximately a 1%-2% prevalence of METex14 skipping mutation, which is found to be very low [19]. METex14 skipping mutations were mostly reported in elderly patients (median age 71.4-76.7 years), usually women, without a smoking history [8,19]. METex14 mutation is considered a genuine oncogenic driver site as it generally occurs in the absence of other driver mutations such as KRAS, EGFR, and HER2 [8,17]. Moreover, METex14 is considered an extremely aggressive subtype with an adverse prognostic factor in patients with NSCLC [8]. The median overall survival (mOS) of anti-MET therapy-naïve patients with stage IV METex14 skipping mutation was found to be 6.7 months, whereas the overall survival (OS) in patients without driver mutations was 11.2 months [20].

Conventional treatment modalities for patients with NSCLC are platinum-based chemotherapy and immunotherapy with immune checkpoint inhibitors (ICIs) [21,22]. However, standard chemotherapy and immunotherapy provide limited benefits to patients with NSCLC harboring METex14 mutation [23,24]. In addition, disease recurrence was reported in patients receiving a combination of immunotherapy and chemotherapy [25].

Targeted therapy using possible oncogenic driver mutations is now an emerging treatment modality [19]. Efficacy and overall response rate (ORR) are found to be higher with targeted therapies than with chemotherapy [19,26]. A study on patients with METex14 skipping mutation reported mOS of 8.1 months (95% confidence interval (CI) = 5.3 to not reported) in those receiving chemotherapy and 24.6 months (95% CI = 12.1 to not reported) in those receiving MET tyrosine kinase inhibitors (TKIs) and the median progression-free survival (mPFS) of MET TKI-treated patients was 7.4 months (95% CI = 3.3 to not reported) [23]. Based on the targeting mechanism, TKIs are categorized into type I MET TKIs and type II MET TKIs. The oral type I MET TKIs are capmatinib, crizotinib, tepotinib, and savolitinib, and type II MET TKIs comprise cabozantinib, merestinib, and glesatinib [27]. Tepotinib, capmatinib, and savolitinib are approved for the treatment of patients with METex14 skipping NSCLC [28,29].

Comprehensive genomic testing that can identify common or uncommon targetable mutations is crucial for the management of patients with metastatic NSCLC [30]. The processes and methods of testing should be dependable to warrant that correct and comprehensive reports timely reach oncologists to initiate appropriate treatment [31]. Nonetheless, in the majority of the Middle Eastern, African, and Russian countries, except Saudi Arabia, national strategies to allow universal access to biomarker testing are lacking. The main constraints of biomarker testing in these countries include performing systematic molecular testing for eligible patients before targeted treatment initiation, tissue availability and its quality, turnaround time, unclear guidelines on biomarkers to be tested, inadequate government or private funding, and lack of support for rare biomarker testing. These unmet needs can adversely affect the diagnosis and management of patients with metastatic NSCLC with METex14 skipping mutation in these countries.

To address these concerns on infrastructural and logistical aspects, a group of expert oncologists from the Middle East, Africa, and Russia was formed to discuss and provide insights into various molecular testing techniques involved in the diagnosis of METex14 skipping mutation in advanced or metastatic NSCLC, management of these patients with targeted therapies, and to develop consensus recommendations for improving these processes while following a modified Delphi method.

## Review

Methodology

An expert multidisciplinary panel consisting of medical oncologists, molecular geneticists, and bioinformaticians from the Middle East, Africa, and Russia was convened to develop consensus statements and/or recommendations for the diagnosis and clinical management of patients with advanced or metastatic NSCLC along with METex14 skipping mutation. Clinically relevant questions associated with NSCLC patients harboring METex14 skipping mutation were developed by prior alignment on relevant questions and key infrastructural recommendations within the international expert group. The objective of this Delphi panel approach was to obtain a more robust, multi-nationally aligned higher quality of applicable recommendations. An electronic survey link to the questionnaire was shared with all participating experts to capture their opinions. The survey results were discussed in the virtual advisory board meeting held on June 18, 2022, and consensus was sought for the relevant questions. A targeted literature search was also conducted to identify the latest evidence on the topics discussed during the meeting, including biomarker testing in METex14 skipping mutation in NSCLC, liquid biopsy versus tissue biopsy, turnaround time, targeted therapy, and so on. The literature search was carried out through PubMed and Google Scholar. English-language publications available from January 2012 to September 2022 were searched by combining the following search/terms using Boolean operators “AND/OR”: “Non-Small Cell Lung Cancer,” “Lung Cancer,” “Oncogene,” “Mutation,” “Driver Oncogene,” “Metastasis,” “MET exon 14 Skipping Mutation,” “Prevalence,” “Immunotherapy,” “Immune Checkpoint Inhibitors,” “Immunomodulators,” “Chemotherapy,” “Targeted Therapy,” “MET Tyrosine Kinase Inhibitors,” “Capmatinib,” “Tepotinib,” “Crizotinib,” “Overall Survival,” “Duration Of Response,” “Treatment‐Related Adverse Events,” “Edema,” “Nausea,” “Hypoalbuminemia,” “Turnaround Time,” “Saudi Arabia,” “Unresectable NSCLC,” “Next-Generation Sequencing,” “Molecular Testing,” “Adenocarcinoma,” “Large Cell Carcinoma,” “Squamous Cell Carcinoma,” “Non-Squamous NSCLC,” “Reverse Transcription Polymerase Chain Reaction,” “Single-Gene Testing,” “Copy Number Variants,” “Single Nucleotide Variants,” “Hybrid-Capture NGS Assay,” “Amplicon-Based NGS Assay,” “MET Amplification,” “First-Line Treatment,” “Second-Line Therapy,” “Maintenance Therapy,” “Liquid Biopsy,” “Tissue Biopsy,” “Circulating Tumor DNA,” “Best Supportive Care,” and “Quality of Life.” After the virtual meeting, the experts reviewed and revised the consensus statements and the recommendations and then agreed to reach the final set of consensus recommendations.

Results and discussion

Section 1: initial workup

Statement 1: All patients with a confirmed NSCLC and staged and identified as unresectable stage IIIB, stage IIIC, or any stage IV should be identified as advanced/metastatic NSCLC based on patient fitness, histology subtyping, and molecular testing before initiating systemic therapy.

The stage of lung cancer at diagnosis has a significant role in treatment outcomes. Early diagnosis can render better survival benefits compared to late-stage diagnosis [32]. Possible surgery, radiation therapy, and chemoradiation therapy are the standard therapies for patients with stage IIIA NSCLC [33,34]. Patients with unresectable stage IIIB and/or IIIC NSCLC are treated based on the tumor site and the performance status (PS) of the patient. Initial chemotherapy, radiation therapy, immunotherapy, or a combination of chemotherapy plus radiation therapy followed by immunotherapy are the treatment modalities for stage IIIB and IIIC NSCLC [33]. However, NSCLC disseminates fast, and about 55% of patients present with stage IV leading to poor prognosis. For stage IV advanced/metastatic NSCLC, the key treatment is systemic therapy (targeted therapy or immunotherapy and/or radiotherapy), as this provides survival benefits and improves disease control and quality of life (QoL). However, it is essential to carefully select suitable patients for systemic therapy with respect to the cancer burden, how much the disease has spread, PS, and other prognostic factors [35].

Recommendations

**Figure 1:**
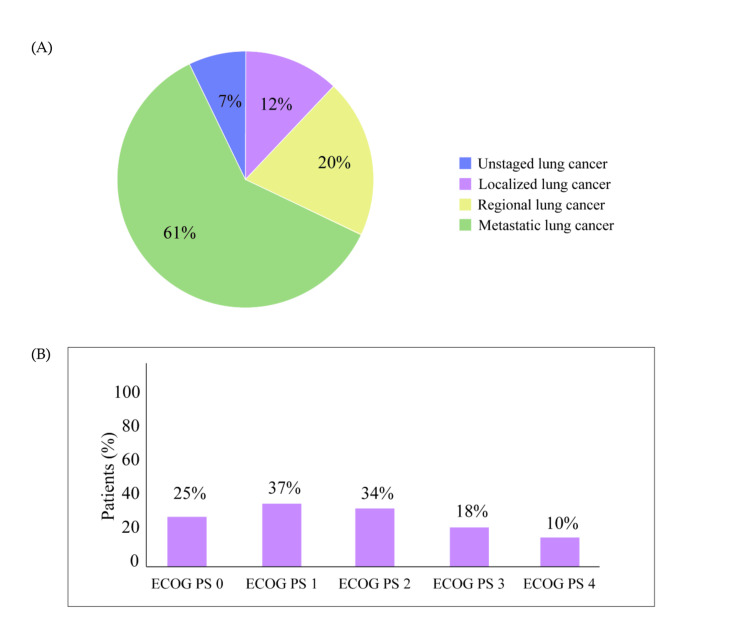
Diagnosis of patients with lung cancer in the Middle East, Africa, and Russia based on the survey result. (A) Spread status of patients with lung cancer during clinical diagnosis. (B) Proportion of patients with NSCLC presenting with ECOG PS 0 to 4. ECOG PS: Eastern Cooperative Oncology Group performance status

Clinicians and physicians should be aware that the majority of patients with NSCLC in the Middle East, Africa, and Russia were diagnosed at metastatic stage (Figure 1A) and presented with Eastern Cooperative Oncology Group Performance Status (ECOG PS) 1 and 2 (Figure 1B). Therefore, failure in molecular testing, lack of sufficient tissue samples, and longer turnaround time can delay treatment initiation in these patients and thereby impact disease management. Patients with poor ECOG PS are particularly not ideal candidates for active therapy. Early assessment of patients with lung cancer along with adequate tissue availability preferably from the primary site for testing is considered the best treatment approach for patients with NSCLC.

Section 2: molecular testing

Biomarker Infrastructure and Logistical Needs

Statement 2: Next-generation sequencing (NGS) molecular testing is recommended for all patients with adenocarcinoma, large-cell carcinoma, NSCLC not otherwise specified, and select cases of squamous cell carcinoma (SCC) (mostly nonsmoker or mixed pathology).

The choice of a suitable molecular test is crucial for the early detection of new as well as already-known biomarkers to decide on the appropriate treatment modalities and improve treatment outcomes. Ideally, the chosen molecular tests should be able to determine all clinically relevant gene modifications, such as insertions or deletions, single-nucleotide variants, gene fusions, and copy number variants [16]. Recently, NGS has emerged as a reliable diagnostic method to detect genetic mutation and copy number alterations in different types of lung cancers [36]. NGS is either a DNA-based or RNA-based assay and is used in large-cell carcinoma, NSCLC, and in NSCLC subgroups, adenocarcinoma and SCC [16,37,38]. Although immunohistochemistry (IHC) is routinely performed for detecting MET overexpression, METex14 skipping mutation cannot be precisely confirmed by IHC. For this mutation, NGS-based molecular testing is a preferred method over IHC, reverse transcription polymerase chain reaction (RT-PCR), and Sanger sequencing [27]. Further, multigene NGS panel sequencing is more cost-efficient and can avoid the exhaustion of tissue samples compared to PCR-based testing [39].

Statement 3: The NGS panel should include targetable genes such as EGFR ex 19 deletion or L858R, EGFR S7681, L861Q, or G719X, KRAS G12C, ALK, ROS 1, BRAF V600E, NTRK 1/2/3, METex14 skipping, RET, PD-L1 with a relative preference to RNA-based testing.

The European Society for Medical Oncology (ESMO) recommends the use of large multigene panel sequencing (MGPS) as several genetic alterations cannot be detected by small gene panels or routine tests [40]. A comprehensive NGS panel, including several targetable genes, is suggested for diagnosis instead of utilizing the single-gene testing option [31]. The main advantage of a comprehensive NGS panel compared to single-gene testing is that a comprehensive NGS panel can detect small insertion/deletions (indels), copy number variants, gene fusions, and single-nucleotide variants. This could be beneficial to laboratories as different variants can be assessed concurrently. The high sensitivity and the ability to incorporate DNA-based and RNA-based assays into a combination system is an added advantage of a comprehensive NGS panel [16]. The molecular testing for MET alterations is recommended to be included in the broad testing panels and not as a single, separate assay. The MET testing should be conducted before treatment initiation or when the test results of ROS1, ALK, and EGFR are negative [41]. In addition, ESMO has currently recommended that in advanced NSCLC cases, molecular testing, including EGFR and BRAF mutations, METex14 skipping mutations, ALK and ROS-1 fusions, RET rearrangements, and PD-L1 expression should be conducted [42].

The NGS can be a DNA-based or RNA-based assay. The DNA-based assay detects genomic variant alterations and ablation of splicing sites, whereas RNA-based assays involve RNA transcript analysis that facilitates the identification of altered splicing and exon 13-15 fusion [9,27]. Numerous RNA splice variants are generated as part of METex14 skipping mutation that may not be captured by the DNA-based assays.

Another major challenge in DNA-based NGS panels is inadequate coverage, as it can detect only 63% of splicing alterations in METex14 variants [27,43]. In contrast, the loss of exon 14 transcription can be directly identified by the NGS RNA sequencing platform making it one of the most conclusive detecting methods [44]. In addition, poor sensitivity is a concern for DNA-based amplicon NGS panels used in detecting METex14 skipping mutations, which can be overcome by RNA-based assays [9]. Hence, DNA-based hybrid-capture or amplicon-based NGS assay should be combined or used along with an RNA-based NGS panel [9,27].

Statement 4: Additional biomarkers such as MET amplifications, genetic alterations, and HER2 mutations may be recommended in patients with failed first-line therapy.

Patients with NSCLC who experienced failure with first-line osimertinib therapy might develop resistance against certain genes leading to treatment failure [45]. A retrospective study by Schoenfeld et al. reported that 19% of patients with first-line osimertinib treatment failure developed off-target resistance to genes, such as MET (amplification), KRAS (mutation), RET, and BRAF (fusion), with the most frequent off-target resistance being MET amplification [46]. MET amplification resistance was also found in patients who were administered MET inhibitor, capmatinib [47]. The HER2 activation is observed in NSCLC through HER2 gene amplification, gene mutation, and protein overexpression [48]. Hence, testing all these biomarkers is essential in patients with first-line treatment failure. The parallel DNA and RNA NGS workflow can detect all genetic alterations. However, there are several challenges associated with the parallel workflow such as the chance of tissue exhaustion, costs, increased turnaround time, and laboratory workload [49].

According to ESMO, large multigene panels can be used based on patient-doctor decisions considering the associated cost and benefits [40]. The MGPS can identify a higher number of patients for targeted therapies compared to single-marker genomic testing (SMGT). However, the MGPS exhibited moderate cost-effectiveness compared to SMGT in patients with advanced-stage IIIB/metastatic NSCLC. Overall, the MGPS versus SMGT incremental cost-effectiveness ratio was detected as $148,478 per life years gained [50]. The increased cost of NGS can be attributed to its reduced availability as a regular diagnostic assessment method in many countries and community settings. Therefore, access to NGS remains unequal globally. Moreover, the use of biomarkers in addition to EGFR, ALK, and ROS1 for patients with NSCLC is a challenge in multiple countries [49]. However, MGPS is generally preferred over SMGT due to the limited availability of tissue and the strenuous process associated with SMGT [51]. The concern of sample exhaustion can also be addressed by MGPS [39].

Specific/National NSCLC Screening Programs

Statement 5: Tissue-based NGS testing is recommended for all patients with adequate tissue biopsy, whereas liquid biopsy-based NGS testing is recommended for patients with inadequate tissue biopsy sample or for patients not responsive to early lines of treatment and who were not tested with a comprehensive NGS panel, including all targetable mutations during their earlier treatments.

NGS-based testing of tissue or liquid biopsy samples can detect METex14 skipping mutation [19,52]. Although tissue biopsy is considered the gold standard for the diagnosis of lung cancer to date and should not be replaced by liquid biopsy, the main limitation of tissue biopsy is the dearth of tumor tissue with nearly 40% of tissue samples deemed limited and inadequate for NGS testing. In addition, about 40% of patients require more than one testing technique to confirm the diagnosis of lung cancer [42].

Currently, liquid biopsy performed using circulating tumor DNA has emerged as an important diagnostic tool for patients with metastatic NSCLC [53]. Liquid biopsies are preferred when tumor tissue is insufficient, inaccessible, or when there is a considerable delay in procuring the tissue [29]. Besides, it can also detect acquired molecular resistance mechanisms in patients with metastatic lung cancer to early lines of targeted therapy [19].

Recommendations

Tissue re-biopsy should be the preferred method of NGS molecular testing over liquid biopsy for lung cancer patients with failed molecular testing unless the patient refuses or a resistant mutation needs to be investigated. Liquid biopsy may be considered complementary to tissue biopsy in case the tissue sample is scanty or inaccessible. Liquid biopsy should be ideally performed before initiating treatment. This could be particularly useful for disease monitoring and prognosis. Molecular testing should be performed before initiating the first line of treatment, and a re-biopsy should be done upon disease progression, preferably tissue biopsy, alone or along with a liquid biopsy.

Section 3: treatment recommendations for NSCLC patients with METex14 skipping mutation

Statement 6: A patient with METex14 skipping mutation and with ECOG PS ≤2 is considered a suitable candidate for treatment, and age should not be a limiting factor for first-line treatment decisions.

Generally, the ECOG PS of a patient is considered by oncologists before treatment initiation. Good PS and minor comorbid conditions are associated with better survival outcomes and QoL in elderly patients receiving chemotherapy/combination chemotherapy. Therefore, ECOG PS is an important factor in a patient’s functional status and needs to be considered while taking treatment decisions in patients with advanced NSCLC [33]. Prominent studies such as VISION and GEOMETRY mono-1 trials were conducted on patients with advanced MET-altered NSCLC with ECOG PS 0/1 as they were considered suitable for treatment [54,55].

Statement 7: Although the preferred and recommended first-line treatment is oral MET-specific TKIs (tepotinib once daily (OD), or capmatinib twice daily (BID)), crizotinib or platinum chemotherapy may be prescribed in case of unavailability or no access to the preferred treatments.

The oral MET TKIs, tepotinib and capmatinib, are recommended by the National Comprehensive Cancer Network (NCCN) Clinical Practice Guidelines in Oncology (NCCN Guidelines®) 2023 as preferred first- and subsequent-line therapies for patients with metastatic NSCLC with METex14 skipping mutation [56]. Tepotinib was approved for the treatment of METex14 skipping mutation. The VISION trial paved the way for the regulatory approval of tepotinib by the US Food and Drug Administration (US FDA) in March 2020. The VISION trial assessed the safety and efficacy of tepotinib in patients with advanced NSCLC with MET alterations [55]. A subgroup analysis of patients with brain metastasis (n = 152) from the VISION trial reported intracranial disease control in 13 out of 15 patients with brain lesions. Partial intracranial best objective response was reported in five of the seven patients who had measurable brain lesions suggesting the durable activity of tepotinib [57]. Capmatinib has evolved as a preferred treatment option for advanced NSCLC with METex14 skipping mutation based on the results from the GEOMETRY mono-1 trial [54].

Although MET TKIs, such as tepotinib and capmatinib, are more selective and potent agents than crizotinib (an ALK-TKI), it was also found to be safe and effective in MET inhibition in patients with advanced METex14-altered NSCLC in PROFILE 1001 trial [58]. The NCCN Guidelines® 2023 has recommended crizotinib as a first-line or subsequent treatment option in certain circumstances for patients with METex14-altered NSCLC [56]. The major clinical trials on patients with advanced NSCLC are summarized in Table 2.

**Table 2:** Key clinical studies on patients with advanced NSCLC. BID: twice daily; CI: confidence interval; ECOG PS: Eastern Cooperative Oncology Group Performance Status; mDOR: median duration of response; MET: mesenchymal-epithelial transition; METex14: mesenchymal-epithelial transition exon 14; mPFS: median progression-free survival; NA: not available; NGS: next-generation sequencing; NSCLC: non-small cell lung cancer; OD: once daily; OS: overall survival; RR: response rate; RT-PCR: reverse transcription polymerase chain reaction

Trial name/Phase/Trial design	Sample size (n)/Patient population	Drug/Dose	Main outcome measures			Adverse events	Clinical implications
			RR (%)/mDOR (months)	mPFS (months)	mOS (months)		
VISION/Phase II/Prospective, open-label, multicenter, single-dose trial [55]	n = 169 Advanced NSCLC with METex14 skipping mutation (ECOG PS 0/1; 0–2 lines of prior therapy)	Tepotinib, 500 mg OD, 9-month cycle	RR: independent review: 46% (95% CI = 36 to 57); investigator assessment: 56% (95% CI = 45 to 66); liquid biopsy: 48% (95% CI = 36 to 61); tissue biopsy: 50% (95% CI = 37 to 63) mDOR: 11.1 months (combined biopsy group; [95% CI = 7.2 to not estimated]), 9.9 months (liquid biopsy group; [95% CI = 7.2 to not estimated]), and 15.7 months (tissue biopsy group; [95% CI = 9.7 to not estimated])	8.5 months (combined biopsy group; [95% CI = 6.7 to 11]), 8.5 months (liquid biopsy group; [95% CI = 5.1 to 11.0]), and 11.0 months (tissue biopsy group; [95% CI = 5.7 to 17.1])	17.1 months (95% CI = 12.0 to 26.8; immature data)	Grade 3 or >3 peripheral edema	The study established tepotinib as a therapeutic option for patients with advanced NSCLC and with a confirmed METex14 skipping mutation as tepotinib led to durable antitumor activity in these patients
GEOMETRY mono-1/Phase II/Multiple-cohort trial [54]	n = 364 Advanced NSCLC (stage IIIB/IV; ECOG PS 0/1; 0–2 lines of prior therapy) with either METex14 skipping mutation or MET amplification	Capmatinib, 400 mg BID, 21 days cycle	Overall response: first- or second-line therapy: 41% (95% CI = 29 to 53); treatment naïve: 68% (95% CI = 48 to 84) mDOR: first- or second-line therapy: 9.7 months (95% CI = 5.6 to 13.0); treatment naïve: 12.6 months (95% CI = 5.6 to not estimated)	First- or second-line therapy: 5.4 months (95% CI = 4.2 to 7.0); treatment naïve: 12.4 months (95% CI = 8.2 to not estimated)	NA	Grade 1-2 peripheral edema, nausea	The study emphasized the need for extensive molecular testing before first-line treatment decisions and concurrent testing with RT-PCR and NGS to detect METex14 alterations in conditions pertaining to limited tissue availability. Also, based on the efficacy and safety profile of capmatinib in this study, it can be considered a new treatment option in advanced NSCLC with METex14 skipping mutation
PROFILE 1001/Phase I/Ongoing, prospective, open-label, multicenter trial [58]	N = 69 METex14 mutated advanced NSCLC	Crizotinib 250 mg BID, 28 days cycle	Objective response rate: 32% (95% CI = 21 to 45) mDOR: 9.1 months (95% CI = 6.4 to 12.7)	7.3 months (95% CI = 5.4 to 9.1)	20.5 months (95% CI = 14.3 to 21.8)	Grade 1-2 edema, nausea, vomiting, vision disorder, diarrhea	The study underscores the need for MET-directed targeted therapy in patients harboring METex14 alterations

However, in case of unavailability of preferred targeted therapy, platinum chemotherapy can be prescribed. The American Society of Clinical Oncology (ASCO) guidelines recommend combination therapy of doublet chemotherapy (platinum/pemetrexed with maintenance pemetrexed) with gefitinib for patients with EGFR-mutant stage IV NSCLC (ECOG PS 0-2) who had not received systemic treatment and under conditions where there is an unavailability of osimertinib [59]. According to the ASCO and Ontario Health (ASCO/OH), nondriver mutation guideline platinum doublet chemotherapy with or without bevacizumab or standard treatment is recommended for patients with exon 20 insertion who develop resistance to first- and second-generation EGFR TKIs [59].

Statement 8: Upon the availability of MET-specific TKIs (tepotinib or capmatinib), patients may be shifted to MET TKIs if treatment is available within two months or all responding patients may be continued on the first-line treatment and MET TKI may be offered in second-line treatment.

As per ASCO/OH nondriver mutation guidelines, targeted therapy with tepotinib or capmatinib or standard therapy is recommended in patients with METex14 skipping mutation as first-line therapy. Subsequently, tepotinib or capmatinib can be considered as second-line therapy for patients with METex14 skipping mutation who were ineligible or received first-line chemotherapy along with or without immunotherapy but were not on first-line MET-targeted therapy [59]. Both VISION and GEOMETRY trials demonstrated the effectiveness of MET-specific TKIs as the first-line treatment than in the second-line settings in improving the median duration of response (mDOR). In the VISION trial, the mDOR was 11.1 months, 9.9 months, and 15.7 months in patients subjected to combined biopsy, liquid biopsy, and tissue biopsy, respectively, while in the GEOMETRY trial, the mDOR in patients on the first- or second-line therapy was 9.7 months and 12.6 months, respectively, in treatment-naïve patients [54,55].

Statement 9: The second-line treatment recommendations for patients with adenocarcinoma, large-cell carcinoma, and NSCLC (ECOG PS 0-1 and PS 2) are pembrolizumab/carboplatin or cisplatin/pemetrexed (category 1) and carboplatin/pemetrexed, respectively, and for patients with SCC (PS 0-1 and PS 2) are pembrolizumab/carboplatin/paclitaxel or pembrolizumab/carboplatin/albumin-bound paclitaxel (category 1) and carboplatin/albumin-bound paclitaxel/gemcitabine/paclitaxel, respectively.

An updated analysis of the open-label, randomized, phase 3 KEYNOTE-024 study found that first-line monotherapy with pembrolizumab improved OS compared with platinum-based chemotherapy in treatment-naïve patients with PDL 1 >50% advanced NSCLC without EGFR/ALK alterations [60] (Table 2). A study on patients with advanced NSCLC who received pembrolizumab monotherapy found that an ECOG PS >2 was associated with a poorer prognosis. This emphasizes the importance of considering ECOG PS on survival outcomes in patients with advanced NSCLC in palliative settings [61].

Statement 10: Only best supportive care (BSC) is recommended for unfit patients with ECOG PS 3-4 with multiple comorbidities and decompensated organ function.

Patients with NSCLC with ECOG PS 3-4 should be provided BSC instead of usual care. Providing palliative care to these patients or those with incurable advanced NSCLC will help to maintain QoL and reduce depression-related psychosocial issues in these patients [7].

Statement 11: For patients who were not initially tested for METex14 skipping mutation and later tested upon first-line treatment failure and identified with METex14, oral MET-specific TKIs (tepotinib OD or capmatinib BID) may be initiated with second-line treatment. In case of disease progression or unacceptable toxicity to MET TKIs, these patients (ECOG PS 0-2) may be switched to ICIs (nivolumab, pembrolizumab, atezolizumab, and no previous immunotherapy) or standard chemotherapy (docetaxel, pemetrexed, gemcitabine, ramucirumab/docetaxel, or paclitaxel, with or without previous immunotherapy).

In case of disease progression after receiving platinum-based chemotherapy, the FDA has approved the use of tepotinib with “breakthrough therapy designation” for second-line therapy in patients with metastatic METex14-altered NSCLC [19]. Patients with advanced sensitive non-T790M EGFR-mutant NSCLC resistant to first/second-generation EGFR-TKI reported moderate activity and good toxicity tolerance when EGFR-TKI was re-administered with afatinib [62]. ICIs such as pembrolizumab, nivolumab, atezolizumab, durvalumab, and avelumab have been extensively used in patients with NSCLC. However, immunotherapy can lead to immune-related adverse events that can be life-threatening. Moreover, combination therapy of programmed death-ligand 1 (PD-L1) ICIs with chemotherapy or targeted therapy can result in the development of toxic reactions [63].

Recommendations

**Figure 2:**
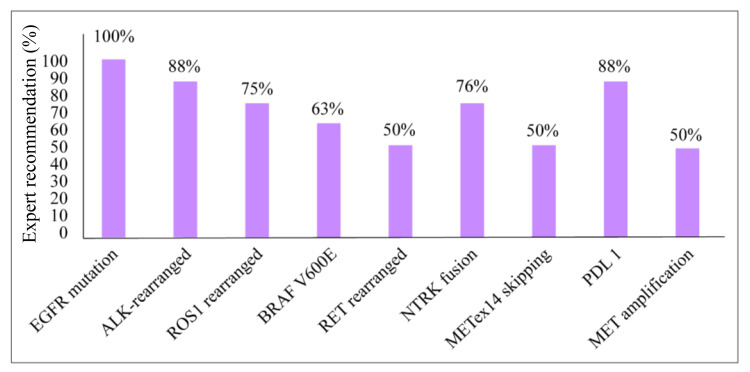
Standard molecular tests for patients with NSCLC in the Middle East, Africa, and Russia based on survey results. ALK: anaplastic lymphoma kinase; BRAF V600E: V-raf murine sarcoma viral oncogene homolog B; EGFR: epidermal growth factor receptor; MET: mesenchymal-epithelial transition; METex14: mesenchymal-epithelial transition exon 14; NSCLC: non-small cell lung cancer; NTRK: neurotrophic receptor tyrosine kinase; PDL 1: programmed death-ligand 1; RET: rearranged during transfection; ROS1: c-ROS oncogene 1

The most common reason/factor for treatment unfitness in patients with NSCLC is considered to be the ECOG PS of the patient rather than the patient’s age. The EGFR mutation, ALK-rearranged, and PD-L1 tests should be the preferred standard tests for lung cancer patients (Figure 2). Initiating a platinum-based chemotherapy regimen and then switching to targeted therapy based on the detection of actionable mutation is recommended for symptomatic NSCLC patients with delayed molecular test results. Immunotherapy with a single agent or in combination with chemotherapy is not recommended even for patients with high PD-L1 expression. Immunotherapy along with targeted therapy might cause severe toxicity in patients with NSCLC. Patients with advanced NSCLC with de novo MET amplification may be offered crizotinib monotherapy and MET inhibitors as the first line and subsequent line of therapies. Selective MET inhibitors were recommended as the first line and subsequent line of therapy for patients with advanced NSCLC harboring METex14 skipping mutations. A regional management recommendation consensus should be developed that can be used as a reference for relatively rare subtypes of lung cancer. Moreover, the panel recommended developing a live regional registry for METex14 NSCLC patients in the Middle East, Africa, and Russia that can serve as a valuable reference specific to this region’s patients.

Section 4: follow-up recommendations

Statement 12: Patients should be monitored for the response after three cycles of chemotherapy by radiology scans and laboratory tests before every cycle to assess the toxicity.

Monitoring of patients after chemotherapy is essential to ensure better clinical outcomes. The NCCN Guidelines 2023 recommend CT scans (with or without contrast) of high-risk sites of the disease after two cycles of chemotherapy followed by every two to four cycles or when clinically indicated. Similar monitoring should be performed for maintenance/subsequent therapy after every six to 12 weeks [56]. Moreover, routine blood tests (red blood cell count, leukocyte count, platelets, neutrophils, and hemoglobin levels) should be performed between chemotherapy cycles to track the treatment-related adverse events for better clinical management [64]. As symptomatic toxicities are common in patients on chemotherapy, the National Cancer Institute’s Common Terminology Criteria for Adverse Events (CTCAE) is considered a standard tool for evaluating toxicity symptoms in patients involved in clinical trials [65].

Recommendations

Patients on chemotherapy should be monitored after two cycles of chemotherapy to evaluate the high-risk sites of the disease. Laboratory tests and CTCAE assessment are recommended for patients before every chemotherapy cycle. Radiological assessment should be performed after three cycles of chemotherapy in patients with good treatment tolerance.

## Conclusions

Most patients with lung cancer are diagnosed at the metastatic stage and the prognosis is poor. Targeted therapy against driver mutation has gained momentum as a promising treatment option for NSCLC. In patients with advanced/metastatic METex14-mutant NSCLC, targeted therapy using MET TKIs demonstrated a better prognosis compared to non-targeted therapy. Performing systematic molecular testing before treatment initiation should be considered a priority for identifying the driver mutation and selecting appropriate targeted therapy. This, in turn, improves the treatment outcomes. Moreover, the consensus recommendations underscored the need for developing regional management recommendation consensus and a live regional registry for patients with METex14 skipping mutation in the Middle East, Africa, and Russia to serve as a valuable reference for managing patients specific to this region.
